# Variation in the OC Locus of *Acinetobacter baumannii* Genomes Predicts Extensive Structural Diversity in the Lipooligosaccharide

**DOI:** 10.1371/journal.pone.0107833

**Published:** 2014-09-23

**Authors:** Johanna J. Kenyon, Steven J. Nigro, Ruth M. Hall

**Affiliations:** School of Molecular Bioscience, The University of Sydney, Sydney, NSW, Australia; Wilfrid Laurier University, Canada

## Abstract

Lipooligosaccharide (LOS) is a complex surface structure that is linked to many pathogenic properties of *Acinetobacter baumannii*. In *A. baumannii*, the genes responsible for the synthesis of the outer core (OC) component of the LOS are located between *ilvE* and *aspS*. The content of the OC locus is usually variable within a species, and examination of 6 complete and 227 draft *A. baumannii* genome sequences available in GenBank non-redundant and Whole Genome Shotgun databases revealed nine distinct new types, OCL4-OCL12, in addition to the three known ones. The twelve gene clusters fell into two distinct groups, designated Group A and Group B, based on similarities in the genes present. OCL6 (Group B) was unique in that it included genes for the synthesis of L-Rhamnose*p.* Genetic exchange of the different configurations between strains has occurred as some OC forms were found in several different sequence types (STs). OCL1 (Group A) was the most widely distributed being present in 18 STs, and OCL6 was found in 16 STs. Variation within clones was also observed, with more than one OC locus type found in the two globally disseminated clones, GC1 and GC2, that include the majority of multiply antibiotic resistant isolates. OCL1 was the most abundant gene cluster in both GC1 and GC2 genomes but GC1 isolates also carried OCL2, OCL3 or OCL5, and OCL3 was also present in GC2. As replacement of the OC locus in the major global clones indicates the presence of sub-lineages, a PCR typing scheme was developed to rapidly distinguish Group A and Group B types, and to distinguish the specific forms found in GC1 and GC2 isolates.

## Introduction

Lipooligosaccharide (LOS) is a lipid-carbohydrate surface structure that is exclusive to the outer membrane of Gram-negative bacteria. Historically the LOS was believed to be essential for outer-membrane stability and survival [1]. However in *Acinetobacter baumannii*, strains lacking LOS have been isolated [2]. None the less, the LOS of *A. baumannii* has been shown to play crucial roles in cell motility [3], surface adhesion [4], resistance to opsonophagocytic killing [5] and to antimicrobial peptides in human serum [6], as well as in the stimulation of the proinflammatory immune response [2], [7]. Modifications of the LOS or its complete loss have also been shown to result in resistance to polymyxin antibiotics such as colistin [2], [8]–[10].

LOS is composed of distinct constituents: the lipid A, which anchors the complex in the outer-leaflet of the outer membrane, and an oligosaccharide. The oligosaccharide consists of an inner core, which is generally conserved in a species, and an outer core (OC) that exhibits variation in the carbohydrate residues it includes and the linkages between them [11]. In Gram-negative bacteria, the genes required to construct the outer core are clustered. In some bacteria, LOS structures can be decorated with a further long polysaccharide (O-antigen) to generate lipopolysaccharide (LPS). However, recent evidence indicates that *A. baumannii* strains form only LOS and a capsule [12]–[17].

**Figure 1:**
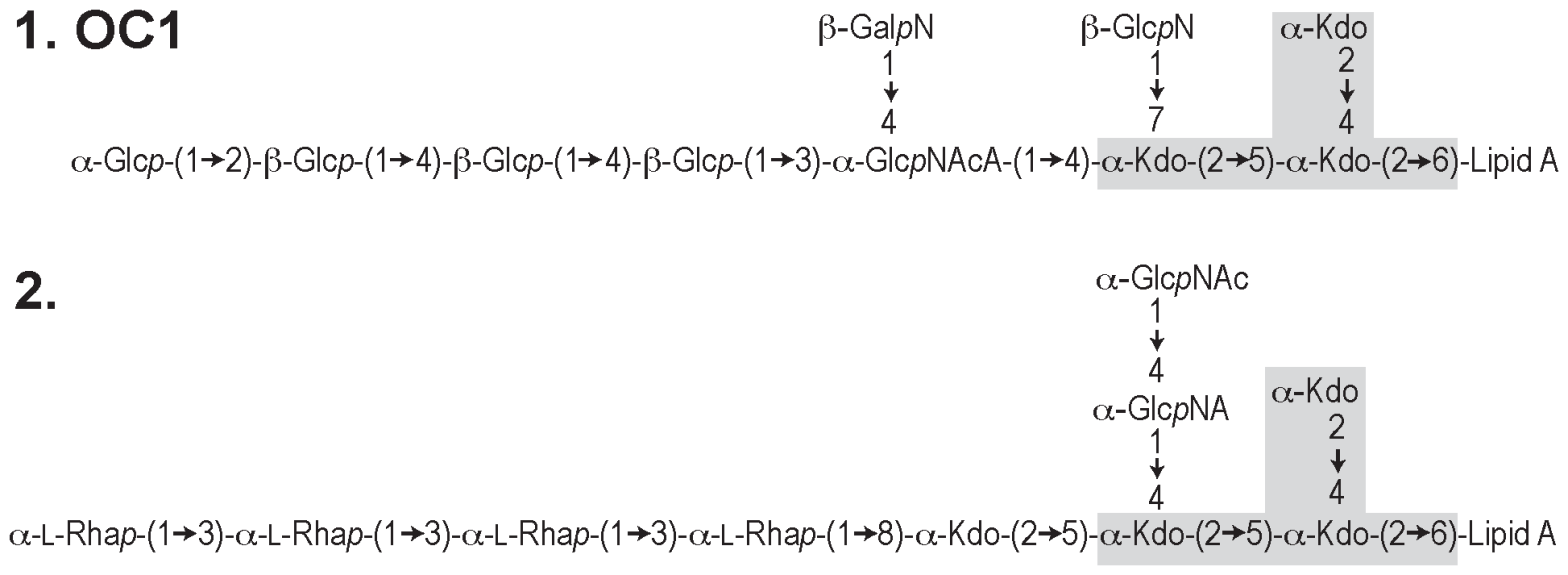
*A. baumannii* solved LOS structures. Structure 1 is OC1 from ATCC 19606 [20] and SMAL [21]. Structure 2 is from ATCC 17904 (NCTC 10303) [25]. Detail of the lipid A portion is not shown. The predicted inner core is shaded in grey. Abbreviations: Kdo, 3-Deoxy-D-*manno*-oct-2-ulosonic acid; Glc*p*, glucose; Glc*p*N, glucosamine; Gal*p*N, galactosamine; Glc*p*NAc, *N*-acetyl-glucosamine; Glc*p*NAcA, *N*-acetyl-glucosaminuronic acid; L-Rha, L-Rhamnose.

The *A. baumannii* chromosome has two clusters of genes for polysaccharide synthesis [12], [18], and one of them clearly represents the K locus for capsule synthesis and export [13], [15], [17], [19]. The second locus, located between *ilvE* and *aspS*, should thus be for the synthesis of the OC component of the LOS. One of only two LOS structures that have been resolved for *A. baumannii*, shown as OC1 in Figure 1, was found in two different isolates, ATCC 19606 [20] and SMAL [21]. We previously found that the draft genome of ATCC 19606 [GenBank accession NZ_GG704577] includes OCL1, and there was a strong correlation between the gene content of OCL1 and the OC1 structure [12]. Recently, the presence of insertion sequences (IS) in three different OCL1 genes was shown to reduce the size of the LOS [16], confirming the role of the OC locus in directing the synthesis of the OC.

**Figure 2:**
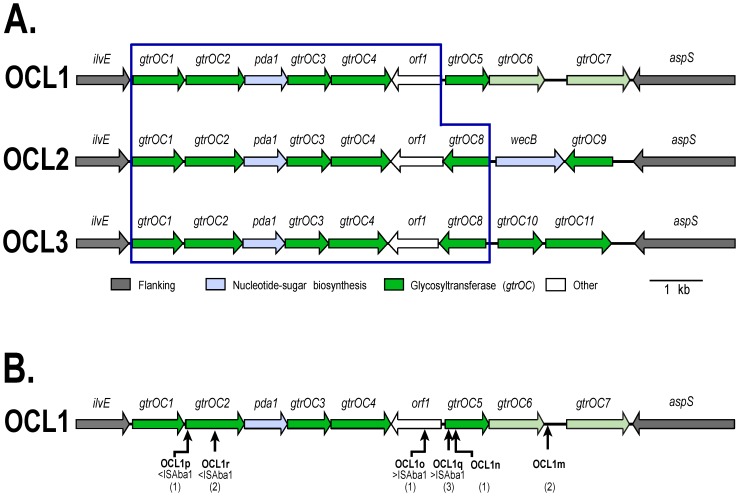
*A. baumannii* OCL1, OCL2 and OCL3 gene clusters. **A**. OCL1, OCL2 and OCL3 arrangements are from [12]. Arrows indicate genes and the direction of transcription. Gene names are shown above, colour scheme key is below, and shared portions are boxed. The gene named *orf1* was *ghy* in [12] (see Table 2). **B**. Location of IS elements in sequenced OCL1 variants. Positions of IS elements are shown below. GenBank accession numbers of OCL1 variants: APBE01000071 and APBF01000005 (OCL1m), APBJ01000096 (OCL1n), ANNC01000028 (OCL1o), AMDQ01000121 (OCL1p), AMIF00000000, AMIG00000000 and AMIU00000000 (OCL1q), AFDO01000006 and ACYS02000025 (OCL1r).

Variation in the OC locus is often seen in Gram-negative species, and three variants, designated OCL1, OCL2, and OCL3, were initially identified in *A. baumannii* genomes [12]. These gene clusters (Figure 2A) were between 11 and 12 kb in length and contained 9 genes, mainly ones that encode the glycosyltransferases that catalyze the linkage of sugars in the OC. The differences between these three forms are in only a small portion consisting of 2–3 genes adjacent to *aspS*. In the previous study, seven of the isolates examined belonged to the two globally disseminated clonal groups, global clone 1 (GC1) and global clone 2 (GC2). These clones account for the majority of extensively antibiotic resistant *A. baumannii* clinical isolates [22]–[24]. OCL1 was seen in 2 of the 3 GC1 isolates, as well as in all 5 of the GC2 isolates examined. However, the third GC1 isolate carried OCL3 suggesting that the gene cluster at the OC locus can be replaced within this clone [12]. More recently, we found OCL1 in 61 Australian GC2 isolates, but 21 Australian GC1 isolates carried either OCL1 (17), OCL3 (3), or a new type, OCL5 (1), that is not related to the other 3 forms [16]. Annotated versions from these sequences can be found in GenBank accessions FJ172370, JN968483, KF130871 and JN247441 for the OCL1 region, and in GenBank accessions KC118540 and HM590877 for OCL3 and OCL5, respectively.

The OC gene cluster responsible for production of the second resolved OC structure that includes several L-Rhamnose*p* residues [25] has not been identified, indicating that further distinct OC gene clusters remain to be discovered. *A. baumannii* is a pathogen of worldwide concern due to the high prevalence of multiply antibiotic resistant isolates causing nosocomial infections, and the present study aimed to explore the full extent of diversity at the OC locus in this important species. 227 draft genomes in the Whole Genome Shotgun (WGS) database and 6 more complete genomes available in GenBank were used to identify new OC forms and to examine the distribution of individual OC locus forms within the species. Variation within the two clonal groups that include most antibiotic resistant isolates can serve as useful epidemiological markers, and here we describe a simple strategy to track sub-lineages of these clones carrying different OC gene clusters.

## Results

### Multi-Locus Sequence Typing (MLST)

The sequence type (ST) was determined for all *A. baumannii* isolates from the sequence data, using the Institut Pasteur MLST scheme (see Methods). The global clones, GC1 and GC2, correspond to sequence types ST1 and ST2 in this scheme, and single locus variants (SLV) were also classified as clone members. Among the draft genomes, 31 were found to be from GC1 isolates (27 ST1, 2 ST19 and 2 ST81) and 100 from GC2 isolates (99 ST2 and 1 ST47). A list of GC1 and GC2 isolates with draft genomes, together with accession numbers, is compiled in Table S1 and Table S2, respectively. A further 51 different STs were found, including ST3 (corresponding to European clone III; Table S3) and 19 new profiles. The genomes examined thus represent a broad range of *A. baumannii* strains suitable for detection of novel OC locus configurations.

### OC locus distribution in GC1 and GC2

As sequencing efforts have focused on the multiply antibiotic resistant isolates belonging to the global clones, the distribution of OC locus forms in these groups was examined first. Four of the additional complete genomes [GenBank accession numbers CP003856, CP003846, CP003849, and AP013357] were from GC2 isolates and these all carried OCL1. Initially, the GC1 and GC2 draft genomes were searched for the presence of the characterized OCL1, OCL2 and OCL3 gene clusters. In all of the GC1 genomes and in 88 of the GC2 genomes, the OC locus was in a single contig or in 2 contigs that could be directly abutted. Most of the GC1 genomes carried OCL1 (9) or OCL3 (14), which had been seen previously. However, OCL2 was also found in 7 isolates, and the new OCL5 form was found in one ST19 isolate, OIFC074 [GenBank accession AMDE01000046] (Table 1). OCL5 is described in detail below. The GC2 chromosomes carried OCL1 (73) or OCL3 (15), indicating that the gene cluster in the OC locus has also been replaced at least once in this clone.

**Table 1:** The distribution of OC locus forms found in multiple STs^a^^,^ b in the draft genomes.

OCL1	OCL2	OCL3	OCL5	OCL6	OCL7	OCL8	OCL9	OCL10
ST1 (8)	ST1 (5)	ST1 (14)	ST19 (1) c	ST6 (1)	ST15 (1)	ST49 (2)	ST218 (1)	ST79 (2)
ST2 (82)	ST10 (2)	ST2 (17)	ST25 (5)	ST9 (1)	ST16 (1)	ST164 (1)	NEW-1 (1)	NEW-7 (1)
ST3 (8)	ST81 (2)^c^	ST136 (1)	ST37 (1)	ST12 (1)	ST113 (2)	ST241 (3)	NEW-4 (1)	
ST11 (1)			ST136 (1)	ST25 (2)	ST221 (1)			
ST19 (1)^c^			ST155 (1)	ST32 (3)	NEW-14 (1)			
ST20 (2)			ST215 (2)	ST33 (1)				
ST47 (1)^d^			ST372 (1)	ST34 (1)				
ST52 (2)				ST36 (1)				
ST78 (4)				ST38 (1)				
ST158 (1)				ST40 (1)				
ST255 (1)				NEW-3 (2)				
NEW-2 (1)^e^				NEW-9 (1)				
NEW-5 (2)				NEW-11 (1)				
NEW-6 (1)				NEW-15 (1)				
NEW-8 (7)				NEW-16 (1)				
NEW-10 (2)				NEW-17 (1)				
NEW-12 (8)								
NEW-13 (1)								

a ST is sequence type.

b Number of isolates of each ST shown in brackets.

c SLV of ST1.

d SLV of ST2.

e NEW is an ST with known alleles but an unrecorded MLST profile.

In the remaining 12 GC2 genomes, the OC locus was interrupted by an insertion sequence (IS), and the final distribution of OCL1 and OCL3 in GC2 is shown in Table 1. The IS was identified by matching the sequence at the ends of the appropriate contigs to sequences available in ISFinder. In 2 isolates carrying OCL3 [GenBank accessions AMSX01000028 and AMHK01000147], ISAba13 interrupted *gtrOC10* at the same position. The locations of IS in the 10 isolates with OCL1 is shown in Figure 2B with the identity of the IS and the number of isolates with a specific insertion indicated below. In those cases where a gene is interrupted, it would be expected that the OC structure will be altered as a consequence, as has been reported recently for other IS insertion mutants [16].

### Distribution of OCL1, OCL2 and OCL3 in other *A. baumannii* genomes

The remaining complete genomes [GenBank accession numbers NC_021733 and AERZ01000079] both included OCL2, and in one of them, NC_021733, an ISAba20 element interrupted the *wecB* gene. Eight draft genomes were from ST3 isolates (Table S3) and all included OCL1. The remaining 96 draft genomes were screened for the presence of OCL1, OCL2 and OCL3. 35 isolates belonging to 15 different STs included OCL1, indicating that OCL1 is very widely distributed (Table 1). Two isolates from ST10 carried OCL2, and a single ST136 isolate carried OCL3.

### Further OC gene clusters

The remaining 53 genomes were examined individually and nine additional OC locus types were found. The open reading frames (ORFs) detected in the new configurations were characterized using Pfam and clan assignments, and these are shown in Table 2. Genes identified were named according to the annotation scheme developed previously (Table 3). Each new locus form was numbered (OCL4-OCL12) in the order identified. The most common were not confined to a specific ST (Table 1). These were OCL5 (12 isolates, 7 STs), OCL6 (20 isolates, 16 STs), OCL7 (6 isolates, 5 STs), and OCL8 (6 isolates, 3 STs). The remaining types were found in only 1 to 3 isolates (Table 1 and Table 4).

**Table 2:** BLASTp, Pfam and Clan Matches searched as of May, 2014.

Protein	GenPept accession	Annotations of BLASTp matches	Pfam	Clan
**Predicted Glycosyltransferases**				
GtrOC1^a^	AGK44465	Hypothetical/Glycosyltransferase	Mito_fiss_Elm1	CL0113
GtrOC2	AGK44464	Glycosyltransferase	Glyco_trans_1_2	CL0113
GtrOC3	AGK44462	Glycosyltransferase	Glycos_transf_2	CL0110
GtrOC4	AGK44461	Glycosyltransferase	Glycos_transf_1, Glyco_transf_4	CL0113, CL0113
GtrOC5	AGK44459	Glycosyltransferase	Glyco_transf_25	CL0110
GtrOC6 b	AGK44458	Hypothetical	n/a	n/a
GtrOC7 b	AGK44457	Glycosyltransferase	n/a	n/a
GtrOC8	AHK10024	Glycosyltransferase	Glyco_transf_25	CL0110
GtrOC9	ABO13302	Glycosyltransferase	Gly_transf_sug	CL0110
GtrOC10	AHK10023	Glycosyltransferase	Gly_transf_sug, Gb3_synth	CL0110, n/a
GtrOC11	AHK10022	Glycosyltransferase	Glycos_transf_1	CL0113
GtrOC12	EPG40646	Glycosyltransferase	Glyco_transf_4, Glycos_transf_1	CL0113, CL0113
GtrOC13	AHK10235	Glycosyltransferase	Glyco_transf_4, Glycos_transf_1	CL0113, CL0113
GtrOC14	AHK10234	Hypothetical	Mito_fiss_Elm1	CL0113
GtrOC15	AHK10233	Glycosyltransferase	Glyco_trans_4_2, Glycos_transf_1	CL0113, CL0113
GtrOC16	AHK10230	Glycosyltransferase	Glyco_transf_8, Glyco_transf_8C	CL0110, n/a
GtrOC17	AHK10229	Glycosyltransferase	Glyco_trans_1_2	CL0113
GtrOC18	EKU52820	Glycosyltransferase	Glyco_transf_4, Glycos_transf_1	CL0113, CL0113
GtrOC19a	EKU52938	Hypothetical/Glycosyltransferase	Mito_fiss_Elm1	CL0113
GtrOC19b	EKP44745	Hypothetical/Glycosyltransferase	Mito_fiss_Elm1	CL0113
GtrOC19c	EKK07863	Hypothetical/Glycosyltransferase	Mito_fiss_Elm1	CL0113
GtrOC20	EKU52965	Glycosyltransferase	Glycos_transf_2	CL0110
GtrOC21	EKU52883	Hypothetical/Glycosyltransferase	Glyco_trans_1_2, DUF3880	CL0113, n/a
GtrOC22	EKP65514	Hypothetical/Glycosyltransferase	Mito_fiss_Elm1	CL0113
GtrOC23	EKP65507	Glycosyltransferase	Glyco_trans_4_2 Glycos_transf_1	CL0113CL0113
GtrOC24	EKP65575	Glycosyltransferase	Glycos_transf_2	CL0110
GtrOC25	EKP65473	Glycosyltransferase	Glycos_transf_1	CL0113
GtrOC26	EKP44834	Glycosyltransferase	Glycos_transf_2	CL0110
GtrOC27	EKK07911	mannosyltransferase	Caps_synth	CL0110
GtrOC28	EKK07913	Glycosyltransferase	Glyco_tranf_2_5	CL0110
GtrOC29	WP_000760350	Glycosyltransferase	Glyco_transf_25	CL0110
GtrOC30	WP_000760349	Glycosyltransferase	Glyco_transf_25	CL0110
GtrOC31	WP_024432893	Glycosyltransferase	Glyco_transf_25	CL0110
**Other**				
Ahy	EKP65391	GDSL-like lipase/acylhydrolase family protein	Lipase_GDSL_2	CL0264
AtrOC1	AHK10232	Acyltransferase	Acyl_transf_3	CL0316
Glf	EPG40651	Glf, UDP-galactopyranose mutase	GLF, NAD_binding_8	CL0063, CL0063
(HtrL)	AHK10231	HtrL/YibB protein	HtrL_YibB	n/a
Orf1 c	AGK44460	Hypothetical protein	n/a	n/a
Orf2	EKP65421	Hypothetical	n/a	n/a
Orf3	WP_000793090	Hypothetical	DUF707	n/a
Pda1	AGK44463	Polysaccharide deaceylase	Polysacc_deac_1	CL0158
Pda2	EKP44902	Polysaccharide deaceylase	Polysacc_deac_1	CL0158
Pda2a	AHK10236	Polysaccharide deaceylase	Polysacc_deac_1	CL0158
PtrOC1	WP_024432894	Glycosyltransferase/pyruvyltransferase/exosortase	PS_pyruv_trans	CL0113
RmlA	EKU52850	RmlA, glucose-1-phosphate thymidylyltransferase	NTP_transferase	CL0110
RmlB	EKU52900	RmlB, dTDP-glucose 4,6-dehydratase	Epimerase	CL0063
RmlC	EKU52972	RmlC, dTDP-4-dehydrorhamnose 3,5-epimerase	dTDP_sugar_isom	CL0029
RmlD	EKU52914	RmlD, dTDP-4-dehydrorhamnose reductase	RmlD_sub_bind	CL0063
WecB	ABO13303	UDP-N-acetylglucosamine 2-epimerase	Epimerase_2	CL0113

a GtrOC1 predicted as a glycosyltransferase as it belongs to Mito_fiss_Elm1 protein family, similar to other GtrOC proteins listed. BLASTp matches hit sequences incorrectly annotated as nucleotide-diphosphate sugar epimerases.

b GtrOC6 and GtrOC7 predicted as glycosyltransferases given that 7 glycosyltransferases are required for construction of OC1 [12]. Preliminary experimental data supports GtrOC6 prediction [16].

c searches conducted in September, 2012 predicted that Ghy belonging to Pfam Glyco_hydro_43 (CL0143) and *ghy* was used in [12]. First BLASTp hit outside of *Acinetobacter* is a putative formyl transferase in *Candidatus Nitrosoarchaeum koreensis* [GenPept accession number WP_007549433] (78% coverage, 37% identity), which also has no predicted Pfam. Gene name has been changed to *orf1* according to updated searches as of May, 2014.

**Table 3:** Key to gene names used.

Gene name	Predicted protein	Predicted reaction product
*ahy*	Acyl- or Acetyl- hydrolase	-
*atrOC*	Acyl- or Acetyl- transferase (outer core)	-
*glf*	UDP-D-galactopyranose mutase	UDP-D-Gal*f*
*gtrOC*	Glycosyltransferase (outer core)	-
*pda*	Polysaccharide deacetylase	UDP-D-Glc*p*N
*ptr*	Pyruvyltransferase	-
*rml*	Multiple	dTDP-L-Rhamnose*p*
*wecB*	UDP-D-Glc*p*NAc C2 epimerase	UDP-D-Man*p*NAc

**Table 4:** OC locus forms found in single draft genomes.

OCL	ST^a.b^	GenBank Accession^c^
OCL4	NEW-18	ASER01000014
OCL11	NEW-19	ASFV01000000
OCL12	ST40	ATGJ01000006

a ST is sequence type – Institut Pasteur scheme.

b NEW is ST with known alleles but an unregistered MLST profile.

c The accession number is for the contig containing the OC locus.

**Figure 3:**
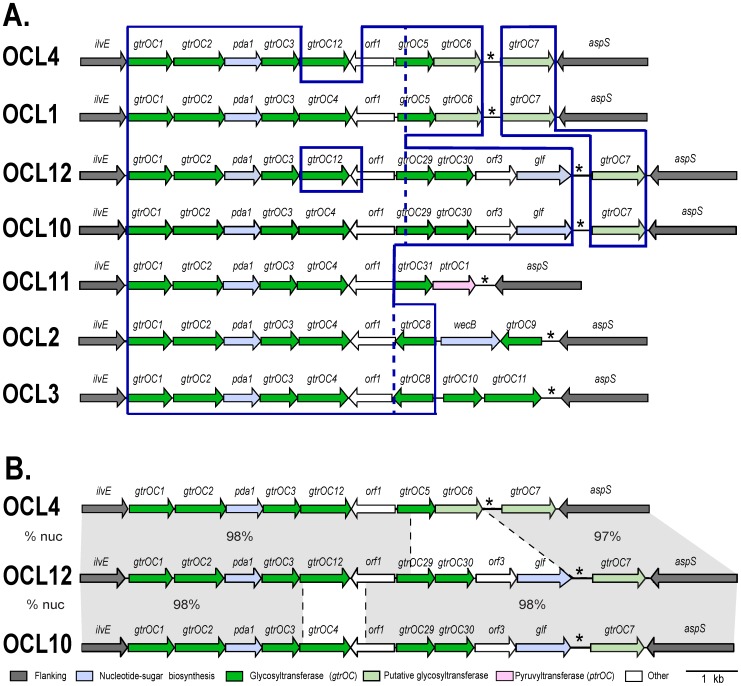
Group A OC locus arrangements. **A**. Genes are shown as arrows indicating the direction of transcription. The colour scheme represents the predicted functional family of the gene products and is shown below. Assigned gene names are shown above as defined in Table 3. Shared regions are boxed. **B**. Comparison of OCL12, OCL4 and OCL10. Grey shading indicates shared regions, with the % nucleotide identity between pairs shown. Figures are drawn to scale from GenBank accession numbers JN968483 (OCL1), CP000521 (OCL2), CP001182 (OCL3), ASER01000014 (OCL4), AMHC01000037 (OCL10), ASFV01000000 (OCL11), and ATGJ01000006 (OCL12).

**Figure 4:**
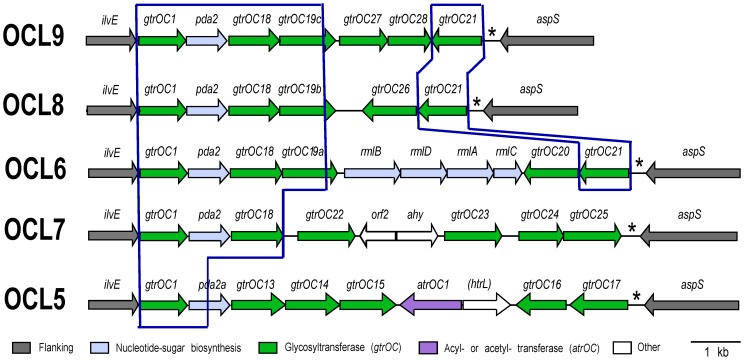
Group B OC locus arrangements. Arrows represent genes showing direction of transcription. Gene annotations are shown above, as defined in Table 3. Modules shared between arrangements are boxed. Colour scheme key is below, and the figure is drawn to scale from GenBank accession numbers HM590877 (OCL5), AMFS01000036 (OCL6), AMTB01000027 (OCL7), ABXK01000027 (OCL8), and ALOH01000183 (OCL9).

OCL1, OCL2 and OCL3 share 6 genes adjacent to *ilvE*, and 4 of the new locus forms were clearly related sharing a minimum of 5 of those 6 genes (Figure 3A). This group was designated Group A. Most of the remaining OC gene clusters formed a separate group, Group B (Figure 4), and OCL5, which appears to be a hybrid with a further type, was most closely related to this group. Although Group B gene clusters are also principally composed of *gtrOC* genes, only the first gene, *gtrOC1*, is shared by both groups suggesting that it may catalyze the first linkage between the inner and outer core. Pairwise nucleotide and protein identities for *gtrOC1* and GtrOC1 of Group A and B are >98%. The differences in genetic composition within and between Groups A and B are described in detail below.

### Group A

Each Group A locus configuration contains between 6 to 8 putative *gtrOC* genes, as well as a *pda1* gene that predicts a polysaccharide deacetylase that enables production of D-galactosamine (D-Gal*p*N) and D-glucosamine (D-Glc*p*N), and an *orf1* gene (*ghy* in [12], see Table 2) previously predicted to encode a glycosyl hydrolase (Figure 3). Four further variants were found but were rare, with OCL10 in 3 isolates (2 ST79) and OCL4, OCL11 and OCL12 in one isolate only. OCL10 and OCL11 share the first six genes with OCL1, OCL2 and OCL3, and all pairwise DNA identities are >98% over this region, but each carry different genes between *orf1* and *aspS*. OCL10 contains an additional nucleotide-linked sugar biosynthesis gene. The *glf* gene predicts a protein with 61% identity to Glf UDP-D-Galactopyranose (UDP-D-Gal*p*) mutase (also known as RfbD) from *Klebsiella pneumoniae* O1 [GenPept accession Q48485] that is required for the conversion of UDP-D-Gal*p* to UDP-D-Galactofuranose (UDP-D-Gal*f*) [26]. OCL10 also contains *gtrOC7* immediately downstream of *aspS*, which extends the similarity to OCL1. However, it is now clear that the 400 bp region marked with an asterisk in Figures 3 and 4 is normally located downstream of *aspS* and that incorporation of *gtrOC7* has separated this region from *aspS.*

Two variants, OCL4 and OCL12, differ from OCL1 and all other Group A members in a 1.25 kb segment that includes *gtrOC12* and ∼260 bp of a *orf1* gene, and replaces *gtrOC4* and the 3′-end of *orf1* (Figure 3A). OCL4 exhibits high identity to OCL1 over the remaining 7.39 kb of the 8.65 kb locus. Likewise, OCL10 and OCL12 share high identity over the majority of the gene cluster, differing only in the 1.25 kb region (Figure 3B). GtrOC4 and GtrOC12 are only 38% identical and, assuming that both enzymes are functional, would be expected to form different linkages possibly between the same sugar substrates. However, the predicted 294 aa Orf1 proteins [GenPept AGK44820 and EPG40647] share 88% identity overall but are >95% identical over the first 208 aa, and 69.7% in the remaining 86 aa.

The OCL12 gene cluster was detected only in the draft genome of a single ST40 isolate, *A. baumannii* strain NIPH 410 [WGS accession ATGJ01000006; GenPept EPG40642 – EPG40653]. The arrangement appears to be a hybrid containing two arms that are much like parts of OCL4 and OCL10 (Figure 3B), sharing >98% identity with their counterparts. OCL4 and OCL12 share the region including *gtrOC1* to *orf1*, and the 5′-end of *gtrOC5* and *gtrOC29*. The first 100 aa of the predicted protein sequences of *gtrOC5* and *gtrOC29* share 95% identity but the other 150 aa are only 44.7% identical. Recombination between OCL4 and OCL10 in approximately 1 kb spanning part of *orf1* and the shared 5′-end of *gtrOC5*/*gtrOC29* would give rise to the OCL12 gene cluster.

### Group B

The OC forms belonging to Group B (Figure 4, Table 5) share a smaller proportion of common sequence adjacent to *ilvE* (2, 3 or 4 genes) than the configurations in Group A. Each Group B form contains 5 to 6 *gtrOC* genes, as well as the *pda2* gene that is predicted to encode a polysaccharide deacetylase (Table 2). Despite being characterized as putative polysaccharide deacetylases based on BLASTp hits and protein family (Pfam) predictions (Table 2), Pda1 (Group A) and Pda2 (Group B) do not share significant sequence identity. In four of the five Group B gene clusters, the *pda2* genes are 99% identical. However, in OCL5, the *pda2a* gene is a hybrid, with the first half (323 bp) 96.7% identical to *pda2* in the other four and the second half (364 bp) significantly diverged (68.1% identity). Hence, OCL5 may be a hybrid with a further form.

**Table 5:** GenPept accessions for representative Group B OC locus forms.

OCL	WGS accession	OCL peptides
OCL5	AFDL01000002	EJG21784 – EJG21756
OCL6	AMFW01000007	ELX01369 – ELX01508
OCL7	AMTB01000038	EKP65593 – EKP65458
OCL8	AMFY01000013	EKP44739 – EKP44897
OCL9	AMFI01000027	EKK07813 – EKK07789

In OCL6, OCL8 and OCL9, the shared portion extends to near the 3′-end of *gtrOC19* with 3 different sequences constituting the remainder of the gene. The three predicted GtrOC19 proteins designated *a*, *b* or *c* share 84–86% identity overall but are 95% identical over the first 283 aa of the total 345 aa indicating that recombination has occurred within this gene on at least two occasions. Despite this, the three GtrOC19 proteins may catalyze the same linkage in the oligosaccharide structures. OCL6, OCL8 and OCL9 also share the last gene in the cluster, *gtrOC21*, and the predicted GtrOC21 proteins are >96% identical. The *gtrOC20* gene in OCL6 and *gtrOC26* in OCL8 are also related but share only 84% DNA identity.

The OCL5 and OCL7 gene clusters include potential sugar modification genes. OCL5 contains an *atrOC* gene predicted to encode an acyltransferase (Table 2). OCL5 also includes a gene that predicts a protein that belongs to the HtrL_YibB protein family, which includes uncharacterized proteins from *Salmonella enterica*, *Escherichia coli* and *Campylobacter jejuni* that are also associated with LOS biosynthesis. OCL7 contains an additional *ahy* gene for a predicted acyl hydrolase.

**Figure 5:**
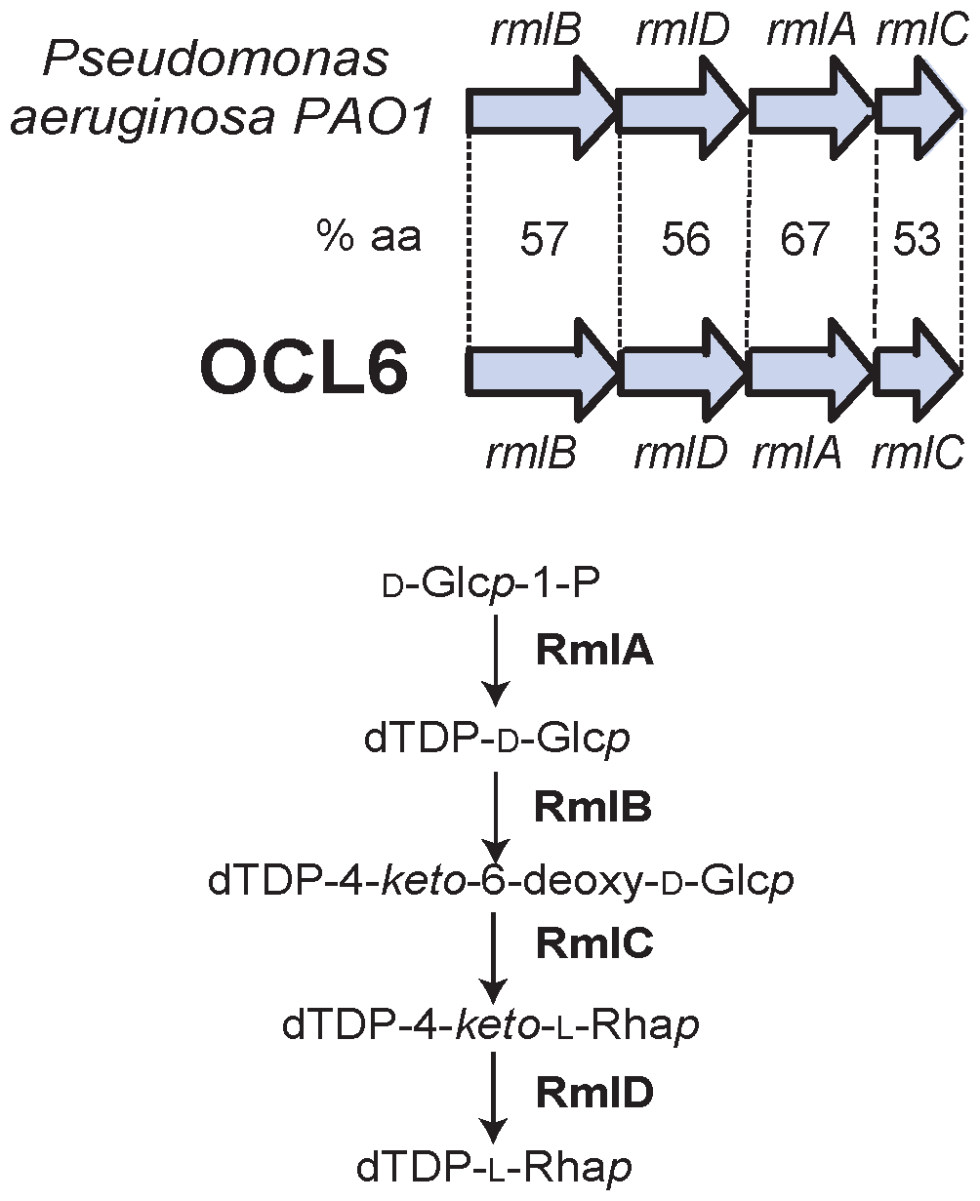
Genes for dTDP-L-Rhamnose synthesis. The *rmlBDAC* gene module from OCL6 [GenBank accession number AMFS01000036] compared to a similar region from *P. aeruginosa* PAO1 [GenBank accession number NC_002516] required for the synthesis of dTDP-L-Rhamnose (dTDP-L-Rha*p*) [27]. The % amino acid identity between predicted gene product pairs is shown. The dTDP-L-Rha*p* biosynthesis pathway is shown below.

OCL6, the second most abundant form found in the genomes analyzed (Table 1), is unique in that it includes a dTDP-L-Rhamnose (dTDP-L-Rha*p*) biosynthesis operon. OCL6 is the only one to contain genes required for the synthesis of a complex sugar precursor. The *rmlB, rmlD, rmlA* and *rmlC* genes are arranged in a module that is located between *gtrOC19a* and *gtrOC20* in OCL6. The four predicted Rml proteins share more than 53% identity with Rml proteins encoded in a *Pseudomonas aeruginos*a PAO1 gene cluster [GenBank accession NC_002516]. In *P. aeruginos*a these genes have been shown to direct the synthesis of dTDP-L-Rha*p* (Figure 5) [27]. The *rml* genes are found in the *rmlBDAC* organization in many bacterial species including *E. coli* and *S. enterica* [28]. The ability to synthesize this sugar suggests that OCL6 may generate the L-Rha*p*-containing OC structure in Figure 1.

### Detection of OC locus variants

**Figure 6:**
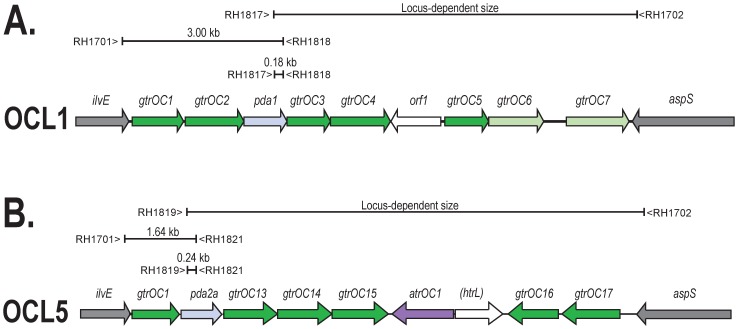
PCR scheme for typing Group A and Group B OC gene clusters. Primer names and positions with PCR product sizes (details in Table 6) are displayed above. **A**. OCL1 represents Group A OC gene cluster forms. **B**. OCL5 for Group B OC gene cluster forms.

Differences at the OC locus have the potential to assist in understanding the epidemiology of *A. baumannii* by detecting variation in otherwise related isolates. Hence, a step-wise PCR approach was developed to facilitate the detection of OC locus types. Short-range PCRs specific for *pda1* or *pda2* were first used to distinguish Group A and Group B gene clusters. To confirm this assignment, the reverse primers in *pda1* or *pda2* were used in conjunction with primer RH1701 to amplify the *ilvE* proximal portion that is conserved in each group (Figure 6, Table 6). Following this, generic long-range PCRs linking either *pda1*-*aspS* or *pda2-aspS* were used to amplify the right arm of the assigned group. As these PCRs yield large products, restriction digestion was used to differentiate specific gene clusters. The PCRs were validated using genomic DNA from sequenced isolates from our own collection that are known to carry OCL1, OCL2, OCL3, and OCL4 for Group A, and OCL5, OCL6, and OCL8 for Group B (data not shown).

**Table 6:** PCR scheme for the detection of OC locus arrangements.

Target groups or forms	PCR type	Target gene(s)	Primers	Sequence (5′ – 3′)	Amplicon size
All	Generic	*ilvE - aspS*	RH1701 RH1702	GCGCACTTGACGGTATTACA GCGCGACTTCAATTCGTGAT	Various
Group A	Generic	*pda1*	RH1817 RH1818	GTGCCCGAGTTTTGCTTATC GCAAGGGCGATACGCATCTG	0.18 kb
Group A	Generic	*ilvE – pda1*	RH1701 RH1818	GCGCACTTGACGGTATTACA GCAAGGGCGATACGCATCTG	3.00 kb
Group A	Subspecific	*pda1 - aspS*	RH1817 RH1702	GTGCCCGAGTTTTGCTTATC GCGCGACTTCAATTCGTGAT	Various
OCL1, OCL4	Subspecific	*ilvE – gtrOC5*	RH1701 RH1704	GCGCACTTGACGGTATTACA CCCTACAAGGTCTTGCCAAT	6.38 kb
OCL1, OCL2, OCL3, OCL10, OCL11	Subspecific	*gtrOC4 - aspS*	RH1705 RH1702	CCTCAGCCCGTACTTACAAC GCGCGACTTCAATTCGTGAT	Various
Group B	Generic	*pda2*	RH1819 RH1820	GGGTCAAGCCCGCTGAGTTT GTCGACCAATCACGATCATG	0.24 kb
Group B	Generic	*ilvE – pda2*	RH1701 RH1821	GCGCACTTGACGGTATTACA GTCGACCAATCACGATCATG	1.64 kb
Group B	Subspecific	*pda2 - aspS*	RH1819 RH1702	GGGTCAAGCCCGCTGAGTTT GCGCGACTTCAATTCGTGAT	Various
OCL1	Specific	*gtrOC4 – gtrOC5*	RH1705 RH1704	CCTCAGCCCGTACTTACAAC CCCTACAAGGTCTTGCCAAT	1.93 kb
OCL2	Specific	*wecB – gtrOC9*	RH1811 RH1812	GAGGAAGCACCAAGTCTGGG CTTTTACTGCTGTTTGGGGC	0.68 kb
OCL3	Specific	*gtrOC10 – gtrOC11*	RH1813 RH1814	GAGGGGGCATGAAGTCCATT GCCAGCGACGTGCAAACCAT	0.99 kb
OCL5	Specific	*gtrOC16 – gtrOC17*	RH1815 RH1816	GCAGCGACACTCCAAGGCTT CCAAACACCAGGTCTGGCAG	1.08 kb

To facilitate the rapid detection of the OC locus types that were found in the majority of isolates belonging to the multiply antibiotic resistant GC1 and GC2 clones, short-range PCRs specific for OCL1, OCL2, OCL3, or OCL5 were also developed (Table 6). The specificity of these PCRs was validated using the OCL representatives listed above (data not shown).

### SMAL carries OCL1

Since SMAL produces the OC1 configuration of LOS [21], the PCR scheme was used to determine if, as predicted, this isolate carries the OCL1 gene cluster. The OCL1-specific PCR (Table 6) detected a product of the expected size, confirming the presence of OCL1 in SMAL. In addition, both the left and right arms were amplified using overlapping PCRs linking *ilvE* to *gtrOC5* (primer RH1701 with RH1704) and *gtrOC4* to *aspS* (primer RH1705 with RH1702) and both reactions yielded products of the expected size. However, given that *gtrOC5* is also present in OCL4, the left-arm PCR was digested with *Xba*I, which yielded fragments of 757, 879, 2009, 2730 bp as predicted for OCL1, whereas OCL4 would produce fragments of 757, 879, 4714 bp.

### Identification of WaaL homologues in non-*baumannii* species

In some Gram-negative bacteria, the complex carbohydrate polymer that is exported as capsule can be also ligated to the LOS as the O antigen to form LPS. This reaction is catalyzed by a WaaL O-antigen ligase. Previously, the absence of a *waaL* gene in 10 complete *A. baumannii* genomes had been determined using BLASTp searches with *Pseudomonas aeruginosa* and *E. coli* WaaL queries [12]. However, two WaaL homologues [GenPept accessions WP_005244245 and WP_010112638] were identified in non-*baumannii* species but their location was not reported. As expected, the *waaL* genes are in the vicinity of the OC locus, located between *aspS* and a gene encoding a TonB-dependent receptor, and separated from *aspS* by a single open reading frame. Here, the BLASTp search was repeated using each of these WaaL sequences as queries. There were no significant hits in any *A. baumannii* derived GenBank entry for which translations were available. However, homologues of WP_005244245 were found in several more recently released genomes of other *Acinetobacter* species (Table 7), and all of them were between *aspS* and *tonB*. The sequence between *aspS* and the TonB-dependent receptor gene was also checked in all *A. baumannii* draft genomes, and no additional genes were found in this location. These findings confirm our previous conclusion that *A. baumannii* strains do not produce LPS.

**Table 7:** WaaL homologues in the vicinity of the OC locus.

Species	GenPept accession	% aa identity to WP_005244245.1 b	% aa identity to WP_010112638.1 b
*Acinetobacter sp.*	WP_005244245.1^a^	100	31
*Acinetobacter sp.*	WP_010112638.1^a^	31	100
*Acinetobacter sp.*	WP_005269248.1	88	29
*Acinetobacter sp.*	WP_016162617.1	79	31
*Acinetobacter sp.*	WP_004802756.1	79	31
*Acinetobacter sp.*	WP_004652452.1	76	31
*Acinetobacter bereziniae*	WP_004827805.1	48	32
*Acinetobacter rudis*	WP_016655105.1	47	31
*Acinetobacter guilouiae*	WP_004723110.1	49	32
*Acinetobacter guilouiae*	WP_004819073.1	48	33

a Reported in [12].

b Identity for proteins with >91% coverage.

## Discussion

Variation at the OC locus has been reported in several Gram-negative species. *E. coli* has five different forms [29], and more extensive variation has been reported for *Campylobacter jejuni* (19 types; [30]) and *Neisseria meningitidis* (11 types; [31]). In this study, nine novel OC gene cluster types were discovered in complete and draft *A. baumannii* genome sequences adding to the three described previously [12], and bringing the total to twelve. This would lead to significant variation in the structure of the LOS on the cell surface, which could affect the various virulence properties associated with LOS in *A. baumannii* [2]–[7]. Additionally, the IS elements found in some OC gene clusters disrupt glycosyltransferase or modification genes and these would also alter the final OC structure.

A notable finding of this study was the identification of a second group of related OC arrangements, Group B. Group B locus forms are unified by shared genes adjacent to *ilvE*, as are Group A forms. Within Groups A and B, there are examples of both small and large regions of sequence difference between pairs of OC configurations. These replacements generally do not include a complete gene or genes. In cases where one gene was replaced with another (*gtrOC4* and *gtrOC12*), the recombination patch extended into adjacent genes, and whether the products of these genes retain their activity or specificity will need to be determined either experimentally or by determining the OC structure. Evidence is mounting to support the conclusion that *A. baumannii* does not produce LPS [12]–[17], and here an additional analysis failed to find a gene for a homologue of WaaL. However, a number of genomes of other *Acinetobacter* species were found to include a predicted *waaL* gene close to their OC locus.

Of twelve different OC gene clusters, OCL1 and OCL6 were the most widespread, with OCL1 in 18 different STs and OCL6 in 16, indicating that recombinational exchange between *A. baumannii* strains is extensive. OCL1 corresponds to one of the solved LOS structures [12]. It is likely that OCL6 directs the synthesis of the second solved structure as it includes L-Rha*p* residues (Figure 1) and only OCL6 contained genes for L-Rha*p* synthesis. Though genes located elsewhere, such as in the locus for capsule synthesis, could contribute this sugar precursor, *rml* genes are found only in a few capsule forms [32]. A link between OCL6 and the solved structure needs confirmation, either by sequencing the OC locus of strain ATCC 17904 [25] or by elucidating the LOS structure of a strain that carries OCL6.

The GC1 and GC2 multiply antibiotic resistant isolates for which genomes were available were from a range of sources, hospitals and countries, but most of them carried OCL1. However, we uncovered further variation at the OC locus in GC1 and for the first time, detected a different OCL in 17 of the 100 in GC2 isolates. OC diversity within the major global clones of *A. baumannii* indicates that this region is being exchanged or replaced repeatedly and more OCL may be found in these clones in the future. To facilitate tracking of these sub-lineages, a comprehensive OCL PCR-typing scheme was developed. Here, it was used to confirm that SMAL carries OCL1. However, this scheme will provide a simple means to distinguish otherwise closely-related strains involved in outbreaks and increase our understanding of the evolution and epidemiology of *A. baumannii*, particularly of the major global clones.

## Materials and Methods

### Bioinformatic analysis

Strain information and accession numbers for the GC1, GC2 and ST3 isolates among the 234 *A. baumannii* genome sequences available on the 1^st^ November 2013 and used in this analysis are listed in Table S1, Table S2 and Table S3. Information on the remaining isolates is in Table 1, Table 4 and Table 5. MLST profiles were extracted from the genomes using a script available at http://sourceforge.net/projects/srst/files/mlstBLAST/, and assigned STs using the Institut Pasteur MLST scheme at http://www.pasteur.fr/recherche/genopole/PF8/mlst/Abaumannii.html.

Genomes containing OCL matches of >97% identity to the full length of OCL1, OCL2 or OCL3, were identified with BLASTn using sequence queries from GenBank accession numbers JN968483 (OCL1), CP000521 (OCL2), and KC118540 (OCL3). Genomes matching parts of all three were assessed for other genes that were identified and characterized as described below. For the remaining genomes, the sequence located between the *ilvE* and *aspS* genes in each genome was located and analyzed as described previously [12]. As the majority of draft genomes available in WGS are unannotated and hence do not include predicted polypeptides, a representative of each novel locus form was assessed for ORFs using the ORF Finder tool (http://www.ncbi.nlm.nih.gov/projects/gorf/). IS elements were identified using the IS Finder database (https://www-is.biotoul.fr//is.html). Proteins predicted from the sequences were analyzed using BLASTp [33] against the non-redundant GenBank database using default parameters, and only cases where polypeptides were co-linear and >25% identical were used for annotation. Predicted protein sequences were also submitted to the protein family (Pfam) database (http://pfam.xfam.org/) [34] and those that included the complete sequence or a domain using default parameters were assigned Pfam classifications. Annotation used an extension of the scheme developed in a previous study [12]. All gene identifiers used in this annotation scheme are summarized in Table 3. Predicted *gtrOC, atrOC* and *pda* sequences were assigned different numbers when the amino acid identity of the gene product was less than 85% to its closest homologue. When IS were present, the OC locus type was considered a variant of a specific OCL and a letter was added after the OCL number.

### PCR

Whole cell DNA was extracted from *A. baumannii* strains as described previously [35]. *A. baumannii* strain SMAL was kindly provided by Cristina De Castro (University of Napoli, Italy).

Primers (Integrated DNA Technologies Inc., San Diego), their targets, and expected amplicon sizes are listed in Table 6. Both short- and long-range PCR amplifications were carried out using genomic DNA as a template. The reaction mix (12.5 µL) for short-range PCR contained 1.25 µL of ThermoPol PCR buffer, 12.5 pmol of each primer, 200 µM of each deoxynucleoside triphosphate, 40 ng of template DNA and 1 unit of *Taq* DNA polymerase (New England BioLabs, Ipswich, MA, USA). Thermocycling conditions were as follows: 94°C for 3 min (initial denaturation cycle), followed by 30 cycles of 94°C for 30 s, 60°C for 30 s and 72°C for 60 s to 3 min, then a final cycle of 72°C for 5 min. For long-range PCR, the reaction mix (20 µL) included 4 µL of HF buffer (Finnzymes, Thermo Fisher Scientific, Australia), 1 µmol of each primer, 200 µM of each deoxynucleoside triphosphate, 40 ng of template DNA and 1 unit of Phusion polymerase (Finnzymes, Thermo Fisher Scientific, Australia). Cycling conditions were 98°C for 30 s (initial denaturation cycle), followed by 35 cycles of 98°C for 10 s, 60°C for 30 s and 72°C for 30 s per 1 kb of expected product size, then a final cycle of 72°C for 10 min.

PCR products were separated by electrophoresis on 1% (w/v) agarose gels and stained with 5 mg/L ethidium bromide. PCR products were visualized with ultraviolet (UV) light and imaged using a GelDoc XR image analysis station (Bio-Rad, Hercules, CA, USA). A 1 kb DNA ladder (New England BioLabs) was used for a molecular size marker. The PCRs were validated using ATCC 19606 (OCL1), ATCC 17978 (OCL2) and AB0057 (OCL3) and isolates from our own collection that carry OCL4 (A388) [23], OCL5 (D13) [16], OCL6 (D46) [36], and OCL8 (J9) [37]. For confirmation of PCR amplicons, products were digested using *Xba*I as per the manufacturers instructions (New England Biolabs).

## Supporting Information

Table S1 **OC forms detected in the draft genomes of** ***A. baumannii*** **ST1 isolates.**
(DOCX)

Table S2 **OC forms detected in the draft genomes of** ***A. baumannii*** **ST2 isolates.**
(DOCX)

Table S3 **OC forms detected in the draft genomes of** ***A. baumannii*** **ST3 isolates.**
(DOCX)
