# Supplementary material for: Variation in the OC Locus of Acinetobacter baumannii Genomes Predicts Extensive Structural Diversity in the Lipooligosaccharide
Source: PLoS One. 2014 Sep 23;9(9):e107833. doi: 10.1371/journal.pone.0107833 (PMC4172580; doi:10.1371/journal.pone.0107833)
Supplement: Table S1 — OC forms detected in the draft genomes of A. baumannii ST1 isolates. (DOCX) [file pone.0107833.s001.docx]

Table S1. OC forms detected in the draft genomes of *A. baumannii* ST1 isolates

| **OCL** | **Strain** | **Accession number** |
| --- | --- | --- |
| OCL1 | AB_TG19582 | AMIV01000075 |
|  | NIPH 290 | APRD01000007 |
|  | NIPH 527 | APQW01000004 |
|  | ABNIH11 | APBA01000015 |
|  | ABNIH19 | APBH01000001 |
|  | ABNIH7 | APAY01000304 |
|  | ABNIH6 | APAX01000029* |
|  | ABNIH10 | APAZ01000078* |
| OCL2 | IS-58 | AMGH01000009 |
|  | IS-235 | AMEI01000029 |
|  | IS-251 | AMEJ01000010 |
|  | AB5075 | AHAH01000026 |
|  | ANC 4097 | APRF01000011 |
| OCL3 | AB_909-02-7 | AMHZ01000020 |
|  | AB_908-13 | AMHW01000070 |
|  | 1605 | AUWL01000012 |
|  | TG22214 | ASFX01000063 |
|  | TG22196 | ASFS01000015 |
|  | TG22194 | ASFR01000022 |
|  | TG22190 | ASFP01000017 |
|  | TG22148 | ASFN01000022 |
|  | TG22112 | ASFK01000001 |
|  | TG20277 | ASFH01000016 |
|  | Canada-BC1 | AMSZ01000013 |
|  | Canada-BC5 | AFDN01000002 |
|  | AB056 | ADGZ01000030* |
|  | AB059 | ADHB01000096* |

* OC locus sequence is found in multiple contigs
