# Supplementary material for: Variation in the OC Locus of Acinetobacter baumannii Genomes Predicts Extensive Structural Diversity in the Lipooligosaccharide
Source: PLoS One. 2014 Sep 23;9(9):e107833. doi: 10.1371/journal.pone.0107833 (PMC4172580; doi:10.1371/journal.pone.0107833)
Supplement: Table S2 — OC forms detected in the draft genomes of A. baumannii ST2 isolates. (DOCX) [file pone.0107833.s002.docx]

Table S2. OC forms detected in the draft genomes of *A. baumannii* ST2 isolates

| **OCL** | **Strain** | **Accession number** |
| --- | --- | --- |
| OCL1 | W7282 | AIEH01000006 |
|  | AB210 | AEOX01000006 |
|  | Ab11111 | AKAQ01000023 |
|  | 53264 | ALPW01000003 |
|  | WM99c | AERY01000087 |
|  | AC12 | ALAM01000007 |
|  | AC30 | ALXD01000010 |
|  | ZWS1122 | AMGR01000003 |
|  | ZWS1219 | AMGS01000006 |
|  | IS-143 | AMGE01000084 |
|  | Naval-113 | AMZU01000067 |
|  | OIFC189 | AFDM01000009 |
|  | AB_1766_8 | AMJO01000195 |
|  | TG22202 | ASFU01000019 |
|  | TG27299 | ASGB01000086 |
|  | TG27315 | ASGE01000015 |
|  | TG27319 | ASGF01000044 |
|  | TG27371 | ASGG01000135 |
|  | TG27379 | ASGH01000072 |
|  | TG27383 | ASGI01000038 |
|  | TG27411 | ASGO01000021 |
|  | TG27407 | ASGN01000035 |
|  | AB_TG27335 | AMIQ01000085 |
|  | AB_TG27331 | AMIP01000025 |
|  | AB_TG27327 | AMIO01000027 |
|  | AB_TG27323 | AMIN01000026 |
|  | TG15233 | ASEV01000103 |
|  | TG15236 | ASEX01000018 |
|  | TG15237 | ASEY01000065 |
|  | TG15238 | ASEZ01000005 |
|  | TG15239 | ASFA01000024 |
|  | TG2013 | ASFF01000016 |
|  | TG2014 | ASFG01000026 |
|  | AB_TG2631 | AMIM01000095 |
|  | AB_TG2026 | AMIH01000412 |
|  | AB_515-8 | AMHU01000096 |
|  | AB_1595-8 | AMHE01000152 |
|  | AB_1582-8 | AMHB01000179 |
|  | AB_908-12 | AMHV01000059 |
|  | AB_909-14-7 | AMIB01000518 |
|  | AB_909-05 | AMIA01000142 |
|  | AB_2009-04-02-7 | AMHT01000084 |
|  | AB_2008-23-07-01-7 | AMHR01000202 |
|  | AB_908-14-7 | AMHX01000291 |
|  | AB_2008-15-45 | AMHL01000074 |
|  | AB_2008-15-70 | AMHO01000234 |
|  | NIPH 528 | APRB01000008 |
|  | NIPH 2061 | APOW01000011 |
|  | NIPH 67 | APRA01000001 |
|  | NIPH 24 | APOF01000004 |
|  | 48055 | AOSP01000135 |
|  | OIFC338 | AMFX01000038 |
|  | ABNIH23 | APBK01000015 |
|  | ABNIH13 | APBB01000003 |
|  | ABNIH14 | APBC01000005 |
|  | Perm | AUZL01000256 |
|  | MRY12-0277 | BASC01000013 |
| **OCL** | **Strain** | **Accession number** |
| OCL1 | MRY09-0642 | BASA01000015 |
|  | MRY10-0558 | BASB01000009 |
|  | AB5711 | AHAJ01000006* |
|  | 3990 | AEOY01000010* |
|  | W6976 | AIEG01000016* |
|  | ABNIH2 | AFTA01000000* |
|  | ABNIH3 | AFTB01000052* |
|  | ABNIH15 | APBD01000085* |
|  | ABNIH20 | APBI01000099* |
|  | ABNIH24 | APBL01000099* |
|  | ABNIH26 | AOGD01000083* |
|  | TG22110 | ASFJ01000026* |
|  | TG22212 | ASFW01000029* |
|  | TG22192 | ASFQ01000073* |
|  | TG2012 | ASFE01000016* |
| OCL1l | Naval-17 | AFDO01000006 |
|  | 6014059 | ACYS02000025 |
| OCL1m | ABNIH16 | APBE01000071 |
|  | ABNIH17 | APBF01000005 |
| OCL1n | ABNIH22 | APBJ01000096 |
| OCL1o | AB1H8 | ANNC01000028 |
| OCL1p | OIFC180 | AMDQ01000121 |
| OCL1q | AB_TG2022 | AMIF00000000 |
|  | AB_TG2023 | AMIG00000000 |
|  | AB_TG5064 | AMIU00000000 |
| OCL3 | TG15242 | ASFD01000068 |
|  | UMB001 | AEPK01000012 |
|  | Ab44444 | AKAT01000029 |
|  | ABNIH25 | APBM01000039 |
|  | Naval-78 | AMFZ01000040 |
|  | ABNIH18 | APBG01000047* |
|  | ABNIH5 | APAW01000088* |
|  | ABIsac_ColiR | CAKB01000002* |
|  | ABIsac_ColiS | CAKA00000000* |
|  | ABNIH1 | AFSZ01000038* |
|  | ABNIH4 | AFTC01000050* |
|  | TG15234 | ASEW01000143* |
|  | TG15240 | ASFB01000104* |
|  | TG20546 | ASFI01000003* |
|  | TG15241 | ASFC01000076* |
| OCL3a | Naval-2 | AMSX01000028 |
|  | 2008-15-34-7 | AMHK01000147 |

* OC locus sequence is found in multiple contigs
