# Supplementary material for: Variation in the OC Locus of Acinetobacter baumannii Genomes Predicts Extensive Structural Diversity in the Lipooligosaccharide
Source: PLoS One. 2014 Sep 23;9(9):e107833. doi: 10.1371/journal.pone.0107833 (PMC4172580; doi:10.1371/journal.pone.0107833)
Supplement: Table S3 — OC forms detected in the draft genomes of A. baumannii ST3 isolates. (DOCX) [file pone.0107833.s003.docx]

Table S3. OC forms detected in the draft genomes of *A. baumannii* ST3 isolates

| **OCL** | **Strain** | **Accession number** |
| --- | --- | --- |
| OCL1 | IS-123 | ALII01000002 |
|  | OIFC109 | ALAL01000012 |
|  | OIFC137 | AFDK01000002 |
|  | NIPH 1669 | APOQ01000003 |
|  | WC-A-694 | AMTA01000004 |
|  | Naval-13 | AMDR01000049 |
|  | Naval-81 | AFDB02000002 |
|  | AB4857 | AHAG01000017 |
